# Parents’ nutrition knowledge, perceived barriers and enablers, and healthy-eating attitudes associated with children’s adherence to the Mediterranean diet: the DELICIOUS project

**DOI:** 10.3389/fnut.2025.1651528

**Published:** 2026-01-05

**Authors:** Sabrina Castellano, Wen Rui Choo, Alice Rosi, Tania Abril Mera, Francesca Scazzina, Francesca Giampieri, Evelyn Frias-Toral, Osama Abdelkarim, Mohamed Aly, Achraf Ammar, Juancho Pons, Laura Vázquez-Araújo, Fernando Maniega Legarda, Lorenzo Monasta, Alessandro Scuderi, Nunzia Decembrino, Ana Mata, Adrián Chacón, Pablo Busó, Giuseppe Grosso

**Affiliations:** 1 Department of Educational Sciences, University of Catania, Catania, Italy; 2 Department of Biosciences and Nutrition, Karolinska Institute, Stockholm, Sweden; 3 Human Nutrition Unit, Department of Food and Drug, University of Parma, Parma, Italy; 4 School of Medicine, Universidad Católica de Santiago de Guayaquil, Tola, Guayaquil, Ecuador; 5 Department of Clinical Sciences, Università Politecnica delle Marche, Ancona, Italy; 6 Research Group on Food, Nutritional Biochemistry and Health, Universidad Europea del Atlántico, Santander, Spain; 7 International Joint Research Laboratory of Intelligent Agriculture and Agri-Products Processing, Jiangsu University, Zhenjiang, China; 8 School of Medicine, Universidad Espíritu Santo, Samborondón, Ecuador; 9 Division of Research, Texas State University, San Marcos, TX, United States; 10 Faculty of Sport Sciences, Assiut University, Assiut, Egypt; 11 ESLSCA University Egypt, Giza, Egypt; 12 Department of Training and Movement Science, Institute of Sport Science, Johannes Gutenberg-University Mainz, Mainz, Germany; 13 Research Laboratory, Molecular Bases of Human Pathology, LR19ES13, Faculty of Medicine, University of Sfax, Sfax, Tunisia; 14 Editorial Luis Vives (EDELVIVES), Carretera de Madrid, Zaragoza, Spain; 15 BCC Innovation, Technology Center in Gastronomy, Basque Culinary Center, Donostia-San Sebastián, Spain; 16 Basque Culinary Center, Faculty of Gastronomic Sciences, Mondragon Unibertsitatea, Donostia-San Sebastián, Spain; 17 Universidade Internacional do Cuanza, Cuito, Bié, Angola; 18 Universidad de La Romana, La Romana, Dominican Republic; 19 Institute for Maternal and Child Health – IRCCS Burlo Garofolo, Trieste, Italy; 20 Department of Agriculture, Food and Environment, University of Catania, Catania, Italy; 21 Neonatal Intensive Care Unit, University Hospital "Policlinico-San Marco" Catania, Integrated Department for Maternal and Child's Health Protection, Catania, Italy; 22 Technological Institute for Children's Products and Leisure AIJU, Alicante, Spain; 23 Department of Biomedical and Biotechnological Sciences, University of Catania, Catania, Italy; 24 Center for Human Nutrition and Mediterranean Foods (NUTREA), University of Catania, Catania, Italy

**Keywords:** Mediterranean diet, children, adolescents, parents, food literacy, nutrition knowledge

## Abstract

**Objective:**

Children’s dietary choices are influenced by several factors, including parents’ modeling. The relation between parents’ psychosocial factors and their children’s level of adherence to the Mediterranean diet were explored.

**Methods:**

Food literacy, perceived barriers and enablers, and healthy-eating attitude following the Capability, Opportunity and Motivation (COM-B) model for behavioral change were evaluated in 2,011 participants in the DELICIOUS (UnDErstanding consumer food choices & promotion of healthy and sustainable Mediterranean Diet and LIfestyle in Children and adolescents through behavIOUral change actionS) project. Adherence to the Mediterranean diet was assessed through the KIDMED questionnaire. Beta coefficients and standard errors (SEs) were calculated through linear regression analyses.

**Results:**

Post-adjustment for potential confounding factors, results showed significant positive correlation between children’s adherence to the Mediterranean diet and parental food literacy [β (SE) = 0.180 (0.011)], perceived barriers and enablers [β (SE) = 0.135 (0.009)], and healthy-eating attitudes (divided into five constructs) [β (SE) = 0.069 (0.030), β (SE) = 0.037 (0.029), β (SE) = 0.162 (0.017), β (SE) = 0.147 (0.010), β (SE) = 0.158 (0.011)]. Individual dietary components of the Mediterranean diet were also associated with various psychosocial factors.

**Conclusion:**

These results confirm the importance of parental food literacy, perceived enablers and barriers to healthy-eating, health-eating attitude in their children’s adherence to the Mediterranean diet.

## 1 Introduction

Over the last decades, current trends of food choice and diet quality in children’s and adolescents have led to the gradual displacement of traditional dietary patterns in favor of unhealthy alternatives (1, 2). One traditional dietary pattern recognised for its health benefits that has been documented to have lost popularity is the Mediterranean diet (3): such a model refers to the traditional eating habits of people in countries bordering the Mediterranean Sea characterized by richness in plant-based foods and moderation in animal ones, with a variety of specific foods, culinary and cultural features that reflect the millenary history of the populations inhabiting such places (4). At its core, the Mediterranean diet emphasizes whole, unprocessed foods that are rich in nutrients, flavor, and cultural significance (5). The Mediterranean diet is based on some key principles. Firstly, an abundance of plant-based foods such as fruits, vegetables, herbs, legumes (e.g., chickpeas and beans), nuts, seeds, and whole grains: these foods are loaded with vitamins, minerals, fiber, and antioxidants (6). Secondly, healthy fats (i.e., monounsaturated fat), such as those from olive oil, a hallmark of the Mediterranean diet (7). Thirdly, moderate consumption of animal-based proteins: the Mediterranean diet advocates for the consumption of fish and seafood over red meat while poultry, eggs, and dairy products are also recommended to be consumed in moderation (8). Next, to enhance the flavor of the food, herbs and spices are preferred sources over salt, sugar, and other ultra-processed condiments; common seasonings observed in the Mediterranean diet are garlic, basil, oregano, and rosemary (9, 10). Lastly, food and beverage aside, an active lifestyle was characteristic alongside the aforementioned features (5). In fact, the Mediterranean diet transcends the limited concept of diet, while it embraces an overall lifestyle that is shaped by culture and tradition passed down from generations before altogether with preference for locally produced foods promoting low environmental impact and health (11, 12). Notably, comprehensive evidence from the scientific literature supports the rationale that higher adherence to the Mediterranean diet would lead to better physical and mental health in both adults and young individuals (13, 14).

Cultivating healthy dietary habits from a young age is crucial as they often persist into adulthood (15, 16). As children are also undergoing rapid growth, a healthy and balanced diet will provide the necessary macronutrients, vitamins, and minerals for proper bone, cognitive, and immune development (17, 18). Several factors, such as family, peers, education, community, television and social media are thought to all play some role in shaping eating behaviors, and they could be perceived as barriers or enablers to the adoption of a healthy diet (19, 20). As home is where children develop their initial knowledge of food, eating attitudes and dietary patterns, parents are thereby examined with the greatest scrutiny as their food literacy and healthy-eating attitude determines the home food environment (including choice of ingredients, food preparation methods and feeding routine) (19). Furthermore, tailored dietary and lifestyle interventions targeting both parents and children have been proven to improve their habits (21).

To influence children’s behavior, there is a need to understand why behavior is as it is and identify what is needed to motivate a change for the desired behavior (22). In this context, a wide range of comprehensive theoretical frameworks encompassing theoretical domains and constructs categorized in various behavior change theories have been applied to investigate psychosocial and behavioral predictors of healthy eating (23). Among the most studied, the Theory of Planned Behavior (TPB) framework posits that the intention to change is the greatest proponent of behavior change (24). The TPB model aims to forecast an individual’s inclination to undertake a specific behavior subject to self-regulation (24). There is therefore a great emphasis on behavioral intent in this model, which evaluates one’s attitude towards the anticipated consequences of the behavior and subjective assessment of the associated risks and benefits (24). However, TPB hinges heavily on the rationality of behavior which may deviate from reality (25). In addition, TPB can be subjective or circumstantial and may not accurately predict behavior (25). The Capability, Opportunity and Motivation Behavior (COM-B) model is another theoretical framework used in the context of behavioral change in nutrition science that has been previously used to identify key determinants of dietary choices and, eventually, for behavioral change (22). The COM-B model suggests that individuals will need the requisite psychological and physical capability (i.e., knowledge and skill to carry out a task), an opportunity defined by social and physical environment enablers (e.g., access to requisite resources and a conducive environment), and the right motivation (e.g., environmental triggers and cultural norms) to engage in a behavior (22). In essence, the COM-B model provides a structured roadmap to break down the various factors that influence human behavior (physical, psychological, social and cognitive) as well as how they work together to elicit and sustain a behavior change (22).

The DELICIOUS (understanding consumer food choices & promotion of healthy and sustainable Mediterranean diet and lifestyle in children and adolescents through behavioral change actions) project is an European project that aims to promote and enable good eating habits among children and adolescents through improving adherence to the Mediterranean diet (26). DELICIOUS is a project involving five different countries in the Mediterranean region – Portugal, Spain, Italy, Egypt, and Lebanon. Recent reports showed that adherence to the Mediterranean diet and diet quality in the participating countries is average (27, 28) and potentially associated with childrens’ lifestyle factors (29). While prior studies have suggested an association between parental attitudes and the dietary choices of their children, no previous harmonized evaluation has been performed across Mediterranean countries concerning parents’ capability, opportunity, and motivation to ensure adherence to the Mediterranean diet in children and adolescents. Therefore, as part of the DELICIOUS project, this study aims to investigate whether parents’ food literacy, their perceived barriers and enablers, and healthy-eating attitude do influence children’s and adolescents’ dietary choices and adherence to the Mediterranean diet in five Mediterranean countries leveraging on the COM-B model as a guiding framework.

## 2 Methods

### 2.1 Study design and participants

For the purposes of this cross-sectional study, a consumer electronic survey was conducted in 2023 among parents of children and adolescents aged between 6 and 17 years old from the five Mediterranean countries involved in the DELICIOUS project. Participants were shortlisted from the consumer database of AIJU Technical Institute for Children’s Products and Leisure and recruited based on their voluntary agreement in being included through an online survey. The inclusion criteria included the following: (i) having children fitting the target population in terms of age range and (ii) having access to the internet to fill out the survey. A target sample size of 400 individuals in each country was deemed adequate to detect significant differences across groups of exposed individuals based on similar studies conducted in Mediterranean countries with similar aims (30–34). However, given the voluntary nature of the participation in the survey, the sample is not considered representative of a random population but, rather, indicative of the target one. A total of 2,011 individuals were finally enrolled in this study. The study protocol was approved by the ethics committee of Mondragon University (no. IEB-20230704). All the procedures were carried out in accordance with the Declaration of Helsinki (1989) of the World Medical Association and all participants signed an informed consent form prior to their participation.

### 2.2 Demographic assessment

Parents were asked to provide their child’s age, gender, height, weight, sleep duration, screen time, and physical activity levels. Body mass index (BMI), defined as weight (in kilograms) divided by the square of height (in meters), was subsequently derived. Unrealistic weight or height values (e.g., <5 kg, or <0.1 m) were excluded from the analysis as they were likely the result of clerical error. Children and adolescents were categorized, according to their age and gender (i.e., BMI z-scores), as either (i) normal weight (BMI 5th–84th percentile), (ii) overweight (BMI 85th–94th percentile), or (iii) obese (BMI ≥ 95th percentile) based on the weight-for-height growth charts percentiles for children and teens ages 2 through 19 years by the Centers for Disease Control and Prevention (CDC) (35). Sleep duration (in hours) was grouped into: (i) <8 h, (ii) 8–10 h, and (iii) >10 h, based on the National Sleep Foundation recommendations for school-age children and teenagers (36). Screen time (in hours) was stratified into: (i) <2 h/day, (ii) 2–4 h/day, and (iii) >4 h/day, based on the Australian 24-h movement guidelines for children and adolescents aged 5 to 17 (37). Finally, physical activity level was assessed using the International Physical Activity Questionnaire-Short Form (IPAQ-SF), which reflects the level of physical activity in the 7 days immediately before the assessment. Specifically, the frequency of activity (measured in days per week) and the duration of activity (time per day) were collected separately for each of the three types of activity (walking, moderate-intensity activity, and vigorous intensity activity), which corresponds to low, medium and high level of physical activity, respectively (38). International standards were referenced, if available. Otherwise, national standards were sourced and referenced as an alternative.

Parents were also asked to provide their age, gender, educational level, family status, family income, and area of living. Educational level was split into: (i) low (primary), (ii) medium (secondary), and (iii) high (tertiary). Family status was categorized as: (i) live alone, (ii) live with a partner, and (iii) live with others. Family income (per month) was stratified into: (i) <€2,000, (ii) €2,000-4,000, and (iii) >€4,000. Finally, the area of living was categorised as: (i) urban, and (ii) rural.

### 2.3 Dietary assessment

A semi-structured 24-h dietary recall and questions on frequency of consumption of major food groups and meal occasions was used to collect data on children’s dietary habits. The level of adherence to the Mediterranean diet was assessed using the Mediterranean diet quality index for children and adolescents (KIDMED) (39). This index is derived from 16 components that summarize the principles of the Mediterranean diet and provides an arithmetic score that ranges from −4 to 12; a score less than 3 reflects a poor diet in relative to the Mediterranean diet principles, whereas values between 4–7 and 8+, average and good adherence to the principles of the Mediterranean diet, respectively. The KIDMED index advocates for a daily consumption of at least one serving of fruit and vegetables, and at least three servings of dairy product: one serving at breakfast and at least two servings of yogurt and/or cheese during the remaining part of the day. Grains and cereals are recommended to be consumed daily at breakfast, whereas pasta or rice should be consumed at least five times per week. At least two to three servings of nuts and fish, and at least two servings for pulses each week are recommended. Olive oil is recommended for culinary use, but frequency is not stipulated. Dietary behaviors that are deemed as detrimental and deviate from the principles of the Mediterranean diet include the frequent intake of sweets and candies (defined as more than twice daily), the consumption of commercially baked goods, pastries and fast-food, and the abstinence from breakfast.

### 2.4 Psychosocial assessment

Referencing the COM-B model, a search of the scientific literature was conducted to identify the assessment tools that could quantify each factor eliciting behavior change (22). The following factors were shortlisted: (i) food literacy which reflects Capability (C), (ii) perceived barriers and enablers which translates to Opportunity (O), and (iii) healthy-eating attitudes and health consciousness which determines Motivation (M) (22).

The Short Food Literacy Questionnaire (SFLQ) for adults was used to measure a broad range of skills including, but not limited to, the functional and interactive elements of the food literacy of parents (40). It is a nine-part questionnaire with a total of 15 items based on a 4- or 5-point Likert scale, where answers may range from “very bad” to “very good,” “strongly disagree” to “strongly agree,” “very difficult” to “very easy,” “very hard” to “very easy,” or “never” to “always.” Of the 15 items, 10 are on the practical skills such as understanding nutrition information and composing a balanced menu, one is on interactive abilities such as exchanging nutrition information with family and peers, and the remaining four assess the ability to critically judge nutrition information or evaluate the long-term impact of dietary habits on health. A maximum score of 62 may be attained, and a higher score indicates higher food literacy.

Perceived barriers and enablers (PBE) were measured through the construction of a set of questions based on the TPB framework positing the intention to change influenced by the following constructs constituting potential barriers and enablers: (i) attitude towards the behavior, which are the personal opinion about the change that may be positive or negative; (ii) subjective norms, which are the community’s (people in the social circle of the subject) opinion about the change that similarly may be favorable or unfavorable; and (iii) perceived behavior control, which refers to the self-perceived level of difficulty to achieve the behavior change (41). Based on earlier studies (42, 43), a three-part questionnaire with multiple questions within each part has been developed based on the TPB model to identify the barriers and enablers of adherence to a Mediterranean diet perceived by the parents. The first part consists of 14 questions and it assesses the attitude towards the behavior. The response is structured based on a five-point Likert scale ranging from one point for “strongly disagree” to five points for “strongly agree” and zero point for the additional “do not know/no answer” option. The second part consists of four questions which assess the subjective norms. Similarly, the response is structured based on a five-point Likert scale ranging from one point for “highly unlikely” to five points for “highly likely” and zero point for the additional “do not know/no answer” option. The final part contains only one question that assesses the perceived behavior control. The response is structured in the same way as the second part. Hence, a maximum score of 95 may be attained.

The Healthy-Eating Attitudes (HEA) questionnaire assesses parent’s attitude towards their child’s diet composition (44). There are a total of 8 questions on parent’s opinions of their child’s intake of fruit, fibre, vegetables, fish, butter, fat, meat, sweets and pastries. The answer to each question is binary (yes or no), where 1 point is allocated for “yes” and 0 point is allocated for “no.” Hence, a maximum score of 8 may be attained.

The Theory of Internet Use related to Health (TIUH) questionnaire is a multiple health domain tool that assesses parent’s tendency to seek health information over the internet to evaluate their health consciousness (45). Four domains are assessed: (i) health-information, which identifies the health-related topics that an individual seeks from the internet, and answers are dichotomous (yes or no); (ii) health consciousness, which consists of five items eliciting a response on a scale of 1 to 5, where “1” representing “strongly disagree,” and “5” representing “strongly agree”; (iii) health-information orientation, which comprises of eight items soliciting a response on a scale of 1 to 5; (iv) health-oriented beliefs, which entails 8 items on the perception of health behavior, and the answers are on a scale of 1 to 5. Hence, maximum scores of 6, 25, 40 and 40 may be attained under the respective domains.

### 2.5 Statistical analysis

Variables were examined for normality and skewness (Kolmogorov and Levene tests). Paired t-test and one-way ANOVA test were used for comparisons of continuous variables according to the normality distribution of the sample. Chi-square test was used to test differences across categorical variables. Univariate linear regression analyses were performed to assess the association between adherence to the Mediterranean diet (calculated as KIDMED score) and scores derived by the assessment tools used to investigate the psychosocial factors within the COM-B model (namely the SFLQ, PBE, HEA, and TIUH). Multivariate linear regression models were additionally performed to assess the relationship between variables of interest (i.e., consumption of certain food groups or compounds, grouped into quantiles) and the outcome by calculating the beta coefficients and standard errors (SEs) adjusted for potential confounding factors, such as children’s age, gender, BMI, physical activity, sleep duration and screen time, parents’ age, gender, education, income, family status, and area of living. *p*-values <0.05 (two-tailed) are considered significant. SPSS 29 (SPSS Inc., Chicago, IL, USA) software was used for all the statistical tests.

## 3 Results

Responses from 2,011 case subjects have been included in this study. An overview of the key demographic profiles of children and adolescents against the parents’ psychosocial assessment scores is in Table 1. The SFLQ scores were higher in the adequate sleep duration group (8–10 h) and higher physical activity level in children whereas the PBE scores were higher for those in the normal weight category, who have adequate sleep duration, and shorter screen time (Table 1).

**Table 1:** Mean scores of parental food literacy (based on SFLQ score), perceived barriers and enablers (PBE score), healthy-eating attitudes (based on HEA score), and healthy-eating behavior (based on TIUH score) by demographic characteristics and lifestyle habits of children and adolescents (*n* = 2,011).

	*n* (%)	SFLQ	PBE	HEA	TIUH			
					Health-Info	Consciousness	Orientation	Beliefs
		Mean score (SD)						
Age								
6–11 y	1,047 (52)	31.9 (5.1)	62.9 (9.0)	7.0 (1.8)	3.4 (2.0)	20.5 (3.2)	31.4 (5.2)	31.1 (6.2)
12–17 y	964 (48)	31.5 (5.0)	63.3 (8.1)	6.8 (1.9)*	3.4 (2.0)	20.4 (3.3)	31.1 (5.3)	31.5 (6.1)
Sex								
Male	995 (49)	31.5 (5.0)	63.5 (8.2)	6.8 (1.9)	3.4 (2.0)	20.4 (3.3)	31.3 (5.3)	31.4 (6.2)
Female	1,016 (51)	31.9 (5.1)	62.7 (8.9)	6.9 (1.8)	3.4 (2.1)	20.5 (3.2)	31.2 (5.2)	31.3 (6.1)
BMI								
Normal weight	1,087 (69)	32.2 (4.9)	64.0 (8.7)	6.9 (1.9)	3.0 (2.0)	20.7 (3.2)	31.1 (5.5)	33.4 (4.8)
Overweight	263 (17)	32.0 (4.9)	62.5 (7.4)	6.8 (1.9)	3.4 (2.1)	20.6 (3.1)	31.3 (5.2)	32.8 (4.9)
Obese	232 (15)	32.4 (5.3)	61.2 (7.7)**	7.4 (1.5)**	4.0 (2.0)**	20.9 (3.1)	32.3 (5.1)*	33.9 (5.1)*
Sleep duration								
<8 h	371 (18)	30.9 (5.2)	62.5 (6.6)	6.7 (2.0)	3.6 (1.9)	20.0 (3.2)	31.0 (5.1)	29.3 (6.3)
8–10 h	1,542 (77)	31.9 (4.9)	63.5 (8.7)	6.9 (1.8)	3.4 (2.0)	20.6 (3.2)	31.3 (5.3)	31.8 (5.9)
>10 h	98 (5)	31.7 (5.8)*	59.3 (9.2)*	7.6 (1.6)**	3.8 (2.1)*	20.3 (3.5)*	32.0 (5.8)	31.2 (7.5)**
Screen time								
<2 h/day	1,131 (56)	31.7 (5.1)	63.8 (8.9)	6.9 (1.8)	3.3 (2.0)	20.5 (3.0)	31.3 (4.8)	31.6 (5.8)
2–4 h/day	723 (36)	31.9 (4.9)	62.6 (8.1)	6.8 (1.9)	3.5 (2.0)	20.3 (3.4)	31.2 (5.7)	31.0 (6.4)
>4 h/day	157 (8)	31.2 (5.7)	61.7 (8.4)*	7.1 (1.7)	3.8 (2.0)*	20.6 (3.5)	31.2 (6.2)	30.8 (7.3)*
Physical activity level								
Low	1,017 (51)	30.9 (5.1)	62.5 (9.0)	6.9 (1.8)	3.3 (2.0)	20.2 (3.2)	31.0 (5.2)	30.9 (6.2)
Medium	461 (23)	32.7 (4.8)	63.6 (7.4)	7.0 (1.8)	3.8 (2.0)	20.8 (3.2)	32.0 (5.1)	32.1 (5.9)
High	533 (27)	32.3 (4.9)**	63.8 (8.6)	6.7 (1.8)	3.3 (2.1)**	20.6 (3.3)*	31.0 (5.5)*	31.5 (6.2)*

BMI, Body Mass Index; SFLQ, Short Food Literacy Questionnaire; PBE, Perceived Barriers and Enablers; HEA, Healthy-eating Attitudes; TIUH, Theory of Internet Use related to Health; y, years. *p < 0.05, **p < 0.001.

On the other hand, the HEA scores showed that parental healthy-eating attitude scores were obese and longer sleep duration groups (Table 1). Moreover, the TIUH scores illustrated that the health-information seeking behavior was stronger in parents of children who are obese, have longer sleep duration, longer screen time, and medium physical activity level. Parents with weaker healthy-eating beliefs are associated with children who are overweight and have low physical activity level, while those with stronger beliefs are associated with children who have adequate sleep duration and shorter screen time (Table 1). An overview of the key demographic profile of the parents against their psychosocial assessment scores is summarized in Table 2.

**Table 2:** Mean scores of parental food literacy (based on SFLQ score), perceived barriers and enablers (PBE score), healthy-eating attitudes (based on HEA score), and healthy-eating behavior (based on TIUH score) by demographic characteristics (*n* = 2,011).

	*n* (%)	SFLQ	PBE	HEA	TIUH			
					Health Info	Consciousness	Orientation	Beliefs
		Mean score (SD)						
Age								
≤44 y	423 (21)	32.5 (5.1)	63.8 (8.2)	7.3 (1.7)	4.5 (1.7)	20.5 (3.3)	32.5 (5.1)	30.8 (6.9)
≥45 y	1,588 (79)	31.5 (5.0)**	62.7 (8.8)	6.8 (1.8)**	3.1 (2.0)**	20.4 (3.2)	30.9 (5.2)**	31.5 (5.9)
Gender								
Male	823 (41)	30.9 (5.0)	60.4 (6.6)	6.8 (1.9)	3.3 (2.0)	20.1 (3.4)	30.7 (5.4)	30.5 (6.5)
Female	1,188 (59)	32.3 (5.0)**	64.2 (9.0)**	6.9 (1.8)*	3.5 (2.0)*	20.7 (3.1)**	31.7 (5.1)**	31.9 (5.9)**
Education level								
Low	91 (5)	28.5 (6.2)	62.3 (7.9)	6.6 (2.3)	3.2 (2.1)	19.4 (4.2)	30/0 (6.6)	28.2 (8.0)
Medium	750 (39)	31.1 (4.8)	65.2 (8.7)	6.8 (1.7)	3.0 (1.9)	20.4 (3.2)	30.7 (5.3)	31.4 (5.7)
High	1,093 (57)	32.5 (4.9)**	62.2 (8.1)**	6.9 (1.8)	3.7 (2.0)**	20.7 (3.1)*	31.8 (5.0)**	31.8 (6.1)**
Family status								
Live alone	249 (12)	31.2 (5.4)	61.4 (8.1)	6.8 (1.9)	3.3 (2.1)	19.8 (4.0)	30.4 (5.8)	30.2 (7.2)
Live with a partner	1716 (85)	31.8 (5.0)	63.5 (8.6)	6.9 (1.8)	3.4 (2.0)	20.6 (3.1)	31.4 (5.1)	31.5 (5.9)
Live with others	46 (2)	30.4 (6.0)*	59.3 (7.4)*	6.3 (1.9)	3.9 (1.6)	19.5 (3.1)**	31.1 (5.3)*	30.5 (7.5)*
Family income								
< €2,000	467 (27)	30.8 (5.2)	63.2 (8.7)	6.8 (1.7)	3.4 (1.9)	20.1 (3.2)	31.1 (5.2)	30.3 (6.2)
€2,000-4,000	796 (47)	32.1 (4.8)	63.7 (8.2)	6.8 (1.8)	3.4 (2.0)	20.5 (3.3)	31.1 (5.3)	32.2 (5.8)
> €4,000	442 (26)	33.0 (4.8)**	62.0 (7.5)*	7.0 (1.9)	4.1 (1.9)**	20.9 (3.1)*	32.4 (5.1)**	32.0 (6.6)**
Area of living								
Urban	1,629 (81)	31.8 (5.1)	62.8 (8.4)	6.9 (1.8)	3.5 (2.0)	20.5 (3.2)	31.3 (5.3)	31.5 (6.1)
Rural	382 (19)	31.3 (4.8)	66.1 (8.9)**	6.8 (1.8)	3.0 (2.0)**	20.4 (3.1)	31.1 (5.0)	30.8 (6.3)

SFLQ, Short Food Literacy Questionnaire; PBE, Perceived Barriers and Enablers; HEA, Healthy-eating Attitudes; TIUH, Theory of Internet Use related to Health; y, years. *p < 0.05, **p < 0.001.

The SFLQ scores were higher in younger mothers with higher educational level, living with a partner and higher family income (Table 2). In contrast, the PBE scores were higher for mothers with mid-range education and income, living with a partner in a rural area (Table 2). However, the HEA scores were similar for most demographic characteristics apart from the age and sex of the parent (Table 2). The TIUH scores were generally higher for young mothers with high education and income who were living with a partner (Table 2). Table 3 shows the association between parents’ psychosocial factors and children’s lifestyle and eating habits.

**Table 3:** Association between parental food literacy (based on SFLQ score), perceived barriers and enablers (PBE score), healthy-eating attitudes (based on HEA score), and healthy-eating behavior (based on TIUH score) and adolescents’ and children’s lifestyle and eating habits.

	SFLQ	PBE	HEA	TIUH			
				Health Info	Consciousness	Orientation	Beliefs
Lifestyle habits							
BMI	0.072 (0.169)	−1.341 (0.359)**	0.225 (0.063)**	0.447 (0.069)**	0.073 (0.109)	0.551 (0.184)*	0.133 (0.166)
Sleep duration	0.504 (0.285)	−0.589 (0.641)	0.372 (0.106)**	−0.021 (0.117)	0.413 (0.184)*	0.593 (0.311)	0.979 (0.281)**
Screen time	−0.168 (0.198)	−1.088 (0.430)*	0.095 (0.073)	0.263 (0.081)**	−0.013 (0.127)	−0.093 (0.216)	−0.002 (0.195)
Physical activity level	0.777 (0.148)**	0.623 (0.341)	0.018 (0.055)	0.03 (0.061)	0.280 (0.095)*	0.187 (0.161)	0.431 (0.146)*
Eating habits							
Breakfast	0.150 (0.178)	1.691 (0.424)**	0.008 (0.064)	−0.323 (0.067)**	0.328 (0.112)*	−0.069 (0.185)	0.711 (0.209)**
Eating with family	0.376 (0.269)	0.513 (0.634)	−0.009 (0.097)	−0.327 (0.101)**	0.330 (0.169)*	0.379 (0.280)	1.034 (0.315)**
Eating alone	0.293 (0.196)	−1.369 (0.486)*	0.173 (0.070)*	0.402 (0.074)**	−0.226 (0.123)	0.170 (0.204)	−0.718 (0.230)*
Eating at school	0.020 (0.139)	−0.729 (0.359)*	0.077 (0.050)	0.237 (0.052)**	−0.066 (0.087)	0.192 (0.144)	−0.194 (0.163)
Eating home-made foods	0.647 (0.174)**	1.305 (0.439)*	−0.128 (0.063)*	−0.403 (0.065)**	0.215 (0.110)*	−0.316 (0.181)	1.394 (0.204)**

KIDMED, Quality Index for Children and Adolescents, SFLQ, Short Food Literacy Questionnaire; PBE, Perceived Barriers and Enablers; HEA, Healthy-eating Attitudes; TIUH, Theory of Internet Use related to Health. *p < 0.05, **p < 0.001.

**Figure 1:**
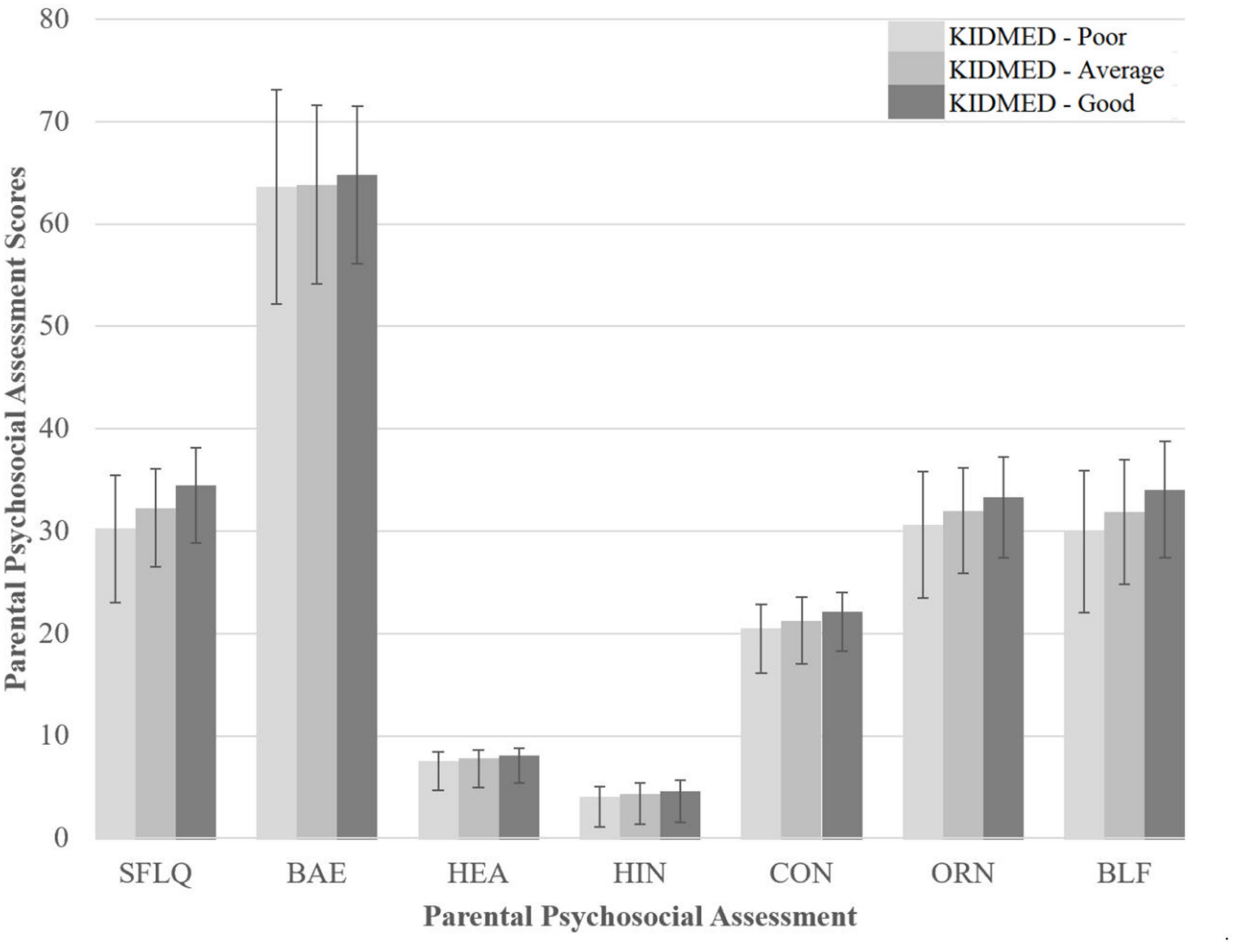
Comparison parental food literacy (based on SFLQ score), perceived barriers and enablers (PBE score), healthy-eating attitudes (based on HEAC score), and healthy-eating behavior (based on TIUH score) by their children’s adherence to the Mediterranean diet (based on KIDMED score). All differences were statistically significant. SFLQ, Short Food Literacy Questionnaire; PBE, Perceived Barriers and Enablers; HEA, Healthy-eating Attitudes; and under the Theory of Internet Use related to Health, there are four components: HIN, Health Information; CON, Consciousness; ORN, Orientation; BLF, Beliefs.

Contrary to expectations, not all constructs were significantly associated with variables of interest and even not in the expected direction. For instance, SFLQ scores predicted higher levels of physical activity; BMI and screen time also decreased with higher scores of PBE; and physical activity levels, breakfast, and family eating habits increased with higher scores on beliefs (Table 3). In contrast, health info was directly associated with BMI and screen time, and inversely with breakfast, eating with family, but also directly with frequency of eating alone and at school (Table 3). Figure 1 provides a graphical illustration of the differences between the psychosocial assessment mean scores across the banded KIDMED scores (i.e., poor, average, and good) resulting in significant differences in all psychosocial constructs analyzed.

The results of the unadjusted and multivariate-adjusted linear regression analyses putting in relation the KIDMED scores with those of the psychosocial assessment scores are summarized in Table 4.

**Table 4:** (PBE score), healthy-eating attitudes (based on HEA score), and healthy-eating behavior (based on TIUH score) and their children’s adherence to the Mediterranean diet (based on KIDMED score).

	Adherence to Mediterranean diet, beta (SE)						
	SFLQ	PBE	HEA	TIUH			
				Health Info	Consciousness	Orientation	Beliefs
Model 1	0.102 (0.009)**	0.023 (0.008)*	0.089 (0.025)**	0.064 (0.023)*	0.101 (0.014)**	0.058 (0.009)**	0.071 (0.007)**
Model 2	0.090 (0.010)**	0.021 (0.008)*	0.106 (0.027)**	0.089 (0.025)**	0.108 (0.016)**	0.067 (0.009)**	0.075 (0.010)**
Model 3	0.090 (0.010)**	0.018 (0.008)*	0.115 (0.027)**	0.125 (0.025)**	0.100 (0.015)**	0.068 (0.009)**	0.071 (0.01)**
Model 4	0.072 (0.011)**	0.028 (0.009)*	0.086 (0.030)*	0.070 (0.029)*	0.090 (0.017)**	0.055 (0.01)**	0.059 (0.011)**

KIDMED, Quality Index for Children and Adolescents; SFLQ, Short Food Literacy Questionnaire; PBE, Perceived Barriers and Enablers; HEA, Healthy-eating Attitudes; TIUH, Theory of Internet Use related to Health. Model 1, unadjusted. Model 2, adjusted for children’s and adolescents’ age, gender, weight status, physical activity, sleep duration, and screen time. Model 3, adjusted for variables in model 2 + breakfast frequency, eating with family, eating alone, eating at school, eating home-made foods. Model 4, adjusted for variables in model 3 + parents’ age, gender, education, income, family status, and area of living. *p < 0.05, **p < 0.001.

After adjusting for various potential confounding factors, all models showed significant results demonstrating a strong association between children’s adherence to the Mediterranean diet and parental food literacy [β = 0.180 (SE 0.011)], perceived barriers and enablers [β = 0.135 (SE 0.009)], healthy-eating attitude [β = 0.069 (SE 0.030)], and health consciousness [for health-info, consciousness, orientation, and beliefs, β = 0.037 (SE 0.029), β = 0.162 (SE 0.017), β = 0.147 (SE 0.010), and β = 0.158 (SE 0.011), respectively] (Table 4). An analysis including the relation between various psychosocial constructs and individual components of the KIDMED showed that, in general, higher scores were in line concerning fruit and vegetable, nuts, and olive oil components, while contrasting associations were found for others and no relation at all for legumes, sweets, and breakfast were retrieved (Table 5).

**Table 5:** Associations between parental food literacy (based on SFLQ score), perceived barriers and enablers (PBE score), healthy-eating attitudes (based on HEA score), and healthy-eating behavior (based on TIUH score) and their children’s adequacy to individual component of the KIDMED score.

	Individual components of the KIDMED, beta (SE)^a^						
	SFLQ	PBE	HEA	TIUH			
				Health Info	Consciousness	Orientation	Beliefs
Fruit, 1 serv/d	1.288 (0.556)*	1.874 (1.569)	0.275 (0.207)	−0.074 (0.221)	−0.193 (0.365)	0.258 (0.591)	0.691 (0.673)
Fruit, 2 serv/d	0.302 (0.254)	−0.909 (0.654)	−0.029 (0.095)	0.292 (0.101)*	0.073 (0.167)	0.195 (0.27)	0.534 (0.308)
Vegetables, 1 serv/d	−0.169 (0.481)	−2.250 (1.225)	0.014 (0.179)	0.364 (0.191)	0.105 (0.315)	0.729 (0.511)	−0.694 (0.582)
Vegetables, 2 serv/d	1.476 (0.248)**	1.029 (0.649)	0.289 (0.092)*	0.178 (0.098)	0.577 (0.162)**	0.963 (0.263)**	0.465 (0.30)
Fish, 2–3 serv/w	0.948 (0.239)**	2.048 (0.616)**	0.025 (0.089)	−0.221 (0.095)*	0.186 (0.157)	−0.130 (0.254)	1.800 (0.290)**
Meat, 1 serv/w	0.238 (0.233)	0.327 (0.587)	0.445 (0.087)**	0.349 (0.093)**	0.358 (0.153)*	1.008 (0.248)**	−0.688 (0.282)*
Legumes, 1 serv/w	−0.350 (0.244)	0.210 (0.633)	0.008 (0.091)	−0.159 (0.097)	0.067 (0.160)	−0.228 (0.259)	0.332 (0.295)
Pasta, 5 serv/w	−0.816 (0.726)	−4.803 (1.782)*	0.147 (0.270)	0.872 (0.289)*	−0.192 (0.476)	0.532 (0.772)	−1.105 (0.878)
Cereals or grains for breakfast	0.278 (0.281)	−0.004 (0.807)	−0.105 (0.104)	0.208 (0.112)	0.077 (0.184)	0.578 (0.298)	−0.761 (0.340)**
Nuts, 2–3 serv/w	1.242 (0.258)**	−0.223 (0.649)	0.138 (0.096)	0.379 (0.103)**	0.519 (0.169)*	1.085 (0.275)**	0.297 (0.313)
Olive oil consumption	2.124 (0.570)**	1.962 (1.652)	0.321 (0.212)	0.483 (0.227)*	0.882 (0.374)*	0.756 (0.606)	4.214 (0.690)**
Skipping breakfast	0.600 (0.602)	−1.251 (1.771)	0.074 (0.224)	0.177 (0.239)	0.315 (0.395)	−0.113 (0.64)	−0.152 (0.729)
Milk or yogurt for breakfast	0.009 (0.257)	4.079 (0.648)**	−0.176 (0.096)	−0.858 (0.102)**	0.363 (0.169)*	−0.343 (0.273)	1.916 (0.311)**
Cakes or pastries for breakfast	−0.869 (0.621)	2.906 (1.596)	0.098 (0.231)	−0.84 (0.247)**	−0.299 (0.407)	−0.871 (0.66)	0.053 (0.751)
Dairy, 2–3 serv/d	1.093 (0.298)**	−1.858 (0.931)*	0.149 (0.111)	0.425 (0.118)**	0.320 (0.195)	1.003 (0.317)*	1.178 (0.36)**
Sweets, several serv/d	−0.884 (0.648)	3.149 (2.026)	−0.149 (0.241)	0.119 (0.258)	−0.422 (0.425)	−0.508 (0.689)	−1.305 (0.784)

SFLQ, Short Food Literacy Questionnaire; PBE, Perceived Barriers and Enablers; HEA, Healthy-eating Attitudes; TIUH, Theory of Internet Use related to Health. *p < 0.05; **p < 0.001.

a Linear regression analyses adjusted for all components of the KIDMED score.

## 4 Discussion

In the present study, the role of parental psychosocial factors related to various aspects of diet and nutrition have been put in relation with their children’s lifestyle and eating habits, ultimately to test whether there was an association with higher adherence to the Mediterranean diet. The results obtained from this study are of particular interest: there were no clear trends between parental psychosocial factors and positive lifestyle and eating outcomes (resulting in mixed results in terms of significance and direction of the associations) but a consistent and strong relation between all psychosocial constructs investigated and higher adherence to the Mediterranean diet was found. Higher food literacy, tendency to seek health information, and beliefs were the constructs associated with the highest number of individual dietary components of the KIDMED score. Recognising the pivotal role that family plays during the formative years of children’s development (46), this study examined how the key attributes of family (in particular parents) are associated with children’s adherence to the Mediterranean diet (46). We have referenced the COM-B model, a robust and comprehensive framework that offers a more holistic approach which considers a wide range of social and environmental influences on behavior taking into account the influence of both internal and external factors (22). Moreover, the COM-B model is dynamic in nature and it acknowledges the synergistic relationship among the three components studied (i.e., capability, opportunity and motivation) (22). More importantly, the COM-B model can provide actionable insights which are constructive for intervention design (22). As a result of its application, a strong association between parents’ food literacy, perceived barriers and enablers, healthy-eating attitude, and health consciousness, and their children’s adherence to the Mediterranean diet was found.

To contextualize our findings against the participants’ demography and uncover potential confounding factors, we also delved into characteristics such as the age, sex, education, income and living arrangements of parents. Considering such factors, younger mothers with higher education and family income have fared better in terms of food literacy, healthy-eating attitude and health consciousness, and their children displayed higher adherence to the Mediterranean diet. Such a profile has been commonly described in previous studies and demonstrated the importance of parental influence in cultivating healthy-eating behavior of young children also in Mediterranean countries (47). There is consistent evidence from the scientific literature relating parents’ education to higher adherence to the Mediterranean diet (48). This finding translates to more specific evidence associating food and nutrition literacy with higher likelihood of adopting a Mediterranean diet, as reported in several studies conducted in Mediterranean countries (49–53). Parents who have the knowledge on the Mediterranean diet and its health benefits will be able to appreciate why their children should adopt the Mediterranean diet and what constitutes a Mediterranean diet. Conversely, failing to understand the effects of diet on health (often perceived as a short-term gain rather than a long-term determinant of health) may lead to an underappreciation of adoption of healthy dietary patterns per se as well as being translated to their children. From the socio-economical point of view, some behavioral constructs significantly differ by parental family income, which are knowingly associated with children’s dietary adherence (54, 55). The relative ease of access of healthy or unhealthy foods, including the availability or the convenience, have also been shown to strongly influence dietary choices and level of adherence to the Mediterranean diet (56).

While previous studies have established an association between various psychosocial factors and dietary adherence, the underlying mechanism remains unclear till date (19). Dietary adherence is a manifestation of human behavior: the cultivation or modification of a human behavior involves a complex confluence of theories from various disciplines, including psychology, sociology, public health and behavioral economics (57). Perceived barriers and enablers have been widely investigated in nutrition science (58). They both play a role in favor or against a trigger for change (22). Children are often nudged and motivated by environmental cues (overt and/or covert) that often come from their parents (59). Hence, parents’ healthy-eating attitude (e.g., opinion on children’s vegetable and fruit intake) and health consciousness (e.g., belief that a healthy diet is the key to disease prevention), which are reflected through words and actions, have a huge influence on children’s dietary choices. From our study findings, we recognise that it is possible to cultivate a behavior (i.e., an adherence to diet) in the presence of the requisite capability (i.e., food literacy), opportunity (i.e., perceived enablers and barriers), and motivation (i.e., healthy-eating attitudes and health consciousness). Every component is critical and they work hand-in-hand to effect a change. The value of the findings shown in the present study relies on the consistent significance of the results across each domain investigated, suggesting the validity of the hypothesis that all components of the COM-B model are implicated when exploring the psychological reasons behind dietary choices (and more specifically in this case, adoption of a Mediterranean diet).

The results presented in this study further investigated also the association between parents’ psychosocial factors with their children’s lifestyle and eating behaviors, revealing that most constructs also predicted overall healthier habits, although not unequivocally. Specifically, while nutrition knowledge and recognizing barriers and enables showed consistent relations with BMI and physical activity, other constructs, such as attitudes and seeking health information on the internet were negatively related with BMI, sleep duration, and other eating habits. While the contrasting findings may underline heterogenous relations, the retrieved results further reinforce the associations accounted for psychosocial factors and adherence to the Mediterranean diet, as those not significant or even resulting in counterintuitive directions are evidently not acting as confounding factors. Other studies fostered evidence for such relations, for instance concerning parents’ nutrition knowledge and children’s physical activity levels (60). Besides, the inconsistency of results concerning those retrieved in relation to seeking information on the internet may be explained by lack or paucity of official portals to retrieved reliable information or, in contrast, exposure to excessive unreliable sources of information, a common well-known issue encountered in other fields, such as vaccination (61).

From a public health perspective, the findings from this study suggest that future studies could include public health nutrition policies leveraging on theoretical framework models, such as the COM-B. Firstly, information should be made more accessible to build capability. Food literacy is a fundamental element as it provides the rationale (i.e., why is a change needed) and roadmap (i.e., how can a change be brought about) for the change (62). For this reason, in Europe, interest in evaluating food literacy within the context of public health has grown, as monitoring this factor can help support professional and policy decisions aimed at improving the well-being of the population (49). Future studies could involve the maternity wards in hospitals and childcare facilities, which are the early touchpoints for young parents, to kickstart education on early feeding practices. Secondly, public infrastructure could be involved to address perceived barriers in order to create opportunity. This may manifest in the form of improving availability, accessibility and affordability of healthy foods. Governments could work with food suppliers (i.e., independent grocers and major supermarket chains) to ensure that healthy food items are well-stocked, physical stores are located at major residential districts, and delivery options are available. In addition, a time-limited subsidy on healthy food items for the lower-income families could also serve as a financial incentive to smoothen the transition. One other hurdle is cultural norms: more effort should be devoted to formulate healthier recipes that respect and preserve the flavors of the local cuisine (i.e., use healthier substitutes – replacing sugar with honey, and high-caloric sauces with herbs). Finally, more environmental cues could be introduced to increase motivation. Public health organizations can consider holding roadshows or campaigns to socialize the population to the concept of healthy eating. Ultimately, the cornerstone of a successful policy is achieving the goal in a palatable and sustainable manner. However, a healthy lifestyle goes beyond diet, it also includes an adequate amount of physical activity. Building on the present study, future studies could examine the factors influencing the level of physical activity in children. Put together, the findings from these two studies have the potential to guide the formulation of an early intervention to elevate the nutritional profile of the children in a sustainable manner.

The findings of this study should be considered in light of its strengths and limitations. This study was conducted in five different countries bordering the Mediterranean Sea. It encompasses representations from South-Western Europe (i.e., Portugal, Spain and Italy), Western Asia (i.e., Lebanon) and North Africa (i.e., Egypt) – the three major coastal regions that demarcate the Mediterranean. Although previous studies have been conducted in these countries, the results shown in the present report are directly multinational in nature sharing the same methodology. This study provides a comprehensive understanding of the factors affecting dietary adherence in the Mediterranean diet taking into account the cultural, economic, and political differences in the three coastal regions. Apart from the good geographical spread, the large sample size also lends greater credibility to the findings of this study. There are also some limitations in this study that are worth mentioning. First, this is a cross-sectional study and therefore its findings cannot be used to establish causality for the associations that are found to be significant. Second, the sample was recruited on a voluntary basis, hence it cannot be considered fully representative of the population although the number of participants is comparable to other studies exploring similar topics. Third, the self-reported data from this study collected through surveys and questionnaires has its inherent limitations. For instance, there may be social desirability bias and memory bias. Also self-rated assessment of psychological factors may be affected by similar limitations. Apart from these biases, which are hard to detect and neutralize, there are genuine clerical errors from self-reporting that renders the response invalid, thereby reducing the sample size. Fourth, data accuracy may also be compromised, referencing KIDMED responses provided by parents. Fifth, there is also a need to recognise and better understand the impact of the wider environment (i.e., extra-familial) on the nutritional status of children, which is not covered in the present study. Such factors include school education, peers and media influences and public infrastructure.

In conclusion, this study has fortified and provided greater clarity to the understanding of parental influence on children’s dietary choices and eventual adherence to the Mediterranean diet. Our results showed a strong positive association among parental food literacy, perceived enablers and barriers to healthy-eating, health-eating attitude, and their children’s adherence to the Mediterranean diet. Investigating the role of family environment, including knowledge, attitudes, and barriers in influencing dietary adherence can also inform more targeted interventions. Studies that delve into the psychological aspects of eating behaviors, including the influence of peer pressure and social media, can provide a more comprehensive understanding of the factors driving dietary choices among young populations. However, studies with an intervention design are needed to properly assess causation of influence of parental psychosocial factors and children’s dietary adherence. The interventions should promote healthy traditional dietary patterns and act on modifiable determinants of adherence, but aiming at improving parents’ nutritional expertise could be crucial to affect their children’s future dietary choices.

## Data Availability

The raw data supporting the conclusions of this article will be made available by the authors, upon reasonable request.
